# Insight into the soil bacterial community succession of *Nicotiana benthamiana* in response to *Tobacco mosaic virus*

**DOI:** 10.3389/fmicb.2024.1341296

**Published:** 2024-01-31

**Authors:** Yuqiang Zhao, Tianbo Liu, Shaolong Wu, Deyong Zhang, Zhipeng Xiao, Zuohua Ren, Lingling Li, Suoni Liu, Yunhua Xiao, Qianjun Tang

**Affiliations:** ^1^College of Plant Protection and College of Bioscience and Biotechnology, Hunan Agricultural University, Changsha, China; ^2^Hunan Tobacco Company, Changsha, China; ^3^Institute of Plant Protection, Hunan Academy of Agricultural Sciences, Changsha, China

**Keywords:** *Tobacco mosaic virus*, bacterial interaction, sensitivity of bacterial community, beneficial bacteria, alpha diversity

## Abstract

**Background:**

*Tobacco mosaic virus* (TMV) is one famous plant virus responsible for substantial economic losses worldwide. However, the roles of bacterial communities in response to TMV in the tobacco rhizosphere remain unclear.

**Methods:**

We explored the soil physicochemical properties and bacterial community succession of the healthy (YTH) and diseased (YTD) plants with TMV infection by 16S rRNA gene sequencing and bioinformatics analysis.

**Results:**

We found that soil pH in the YTD group was significantly lower than in the YTH group, and the soil available nutrients were substantially higher. The bacterial community analysis found that the diversity and structure significantly differed post-TMV disease onset. With TMV inoculated, the alpha diversity of the bacterial community in the YTD was markedly higher than that in the YTH group at the early stage. However, the alpha diversity in the YTD group subsequently decreased to lower than in the YTH group. The early bacterial structure of healthy plants exhibited higher susceptibility to TMV infection, whereas, in the subsequent stages, there was an enrichment of beneficial bacterial (e.g., *Ramlibacter*, *Sphingomonas*, *Streptomyces*, and *Niastella*) and enhanced energy metabolism and nucleotide metabolism in bacteria.

**Conclusion:**

The initial soil bacterial community exhibited susceptibility to TMV infection, which might contribute to strengthening resistance of Tobacco to TMV.

## Introduction

*Tobacco mosaic virus* (TMV) is the first virus to be discovered and identified, a single-stranded RNA (Creager et al., 1999). The optical temperature for TMV proliferation is 25–30°C, and its reproduction is inhibited at above 37°C. TMV can infect more than 400 species of plants in 36 families, such as Cruciferae, Solanaceae, Compositae, and Chenopodium (Scholthof et al., 2011). It is also a common tobacco virus disease and can remain in the soil to become the primary infection source for the next year after the winter (Hossain et al., 2022). Field infection is mainly via sap transmission. The gentle friction between the sick and healthy leaves causes damage, and the virus can invade through micro-wounds. Secondly, human activities and chewing mouth insects (e.g., locusts and tobacco green worms) can spread the virus. During tobacco cultivation, the incidence rate of TMV ranges from 20 to 30%, with some instances reaching as high as 60–80%, resulting in significant economic losses for the industry (Islam et al., 2018).

The commonly used methods mainly include chemical pesticides and transgenic approaches, which cannot eliminate TMV load in infected tobacco plants and whose efficacy is still limited in the field. Microbes (e.g., *Streptomyces*, *Pseudomonas*, and *Bacillus*) have been considered potential alternatives for chemical pesticides to inhibit plant pathogens, including fungal, bacterial, and viral pathogens (Kim et al., 2022; Ren et al., 2022; Shi et al., 2022). *Pseudomonas fluorescens* could inhibit the activity of *Cucumber mosaic virus*, *Tomato mottle virus*, and *Tobacco necrosis virus* (Maurhofer et al., 1998; Murphy et al., 2000). *Azotobacter vinelandii*, and *Bacillus subtilis* could inhibit *Potato virus*, *Potato leaf roll virus*, and *Potato virus* Y in *Solanum tuberosum* (Beris et al., 2018). The study of Tollenaere et al. (2017) found that the bacterium *Xanthomonas oryzae* decreased the relative estimate of rice yellow mottle virus load by about 50%. As a result, it is essential to note that regulating bacterial communities and increasing beneficial bacteria could influence plant resistance to TMV.

Currently, numerous bacterial strains have been employed to prevent TMV infection. For instance, Damayanti and Katerina (2008) found that *B. cereus* (I–35) and *Stenotrophomonas* sp. (II-10) can reduce the infection of TMV in hot pepper. Lian et al. (2011) found that *Rhodopseudomonas palustris* GJ-22 could inhibit TMV in *Nicotiana tabacum*. In addition, *P. chlororaphis* O6N was resistant to TMV in *N. tabacum* cv Xanthi-nc (Park et al., 2012).

Many studies have focused on comparing the differences in soil microbial communities between diseased and healthy plants (Liang et al., 2021; Hossain et al., 2022; Tang et al., 2023). However, there is still limited research on the response mechanism of soil bacterial communities and plants to the pathogen attack (Lu et al., 2022; Kuang et al., 2023). In this study, we explored the succession of soil bacterial communities of the healthy and diseased plants after *N. benthamiana* inoculated with TMV to indicate the role of soil bacterial communities in controlling TMV and the potential beneficial microbes.

## Materials and methods

### Pot experiment and virus inoculation

The pot soils were obtained from the 0 to 20 cm depth layer at the Yunyuan test site of Hunan Agricultural University in Changsha, China. The soils were naturally air-dried and passed through a 5 mm sieve for the pot experiment. The properties of pot soil were as follows: the pH value was 5.32, and the contents of organic matter (OM), total nitrogen (TN), nitrate nitrogen (NO_3_^–^-N), ammonium nitrogen (NH_4_^+^-N), total phosphorus (TP), available phosphorus (AP), total potassium (TK), available potassium (AK) were 97.500, 3.790, 0.034, 0.108, 1.269, 0.046, 15.200, and 0.214 g/kg, respectively.

The healthy *N. benthamiana* seedlings (5–7 true leaves) were transplanted into the 40 pots (15.5 cm × 14 cm) containing 1.0 kg soil. One plant was transplanted in each pot. Fifteen days after transplantation, we rinsed 80 ml TMV suspensions into each plant root. TMV suspensions (wt: vol, 1:30) were prepared by abrasing infected tobacco leaves with carborundum abrasive powder and phosphate buffer (pH 7.0). No pest or disease controls were applied during the experiment. Soil samples (5.0 g) were collected from the vicinity of the roots (2–4 cm) on days 7, 14, 21, and 28 after viral exposure to the plants. The collected samples were then stored at −80°C. On day 28, the incidence TMV was assessed in accordance with the Chinese national standard (GB/T23222-2008) and molecular detection of tobacco leaves using a specific TMV primer (Kumar et al., 2011). After inoculating TMV, there were seven among 40 tobacco plants to be healthy, and 33 were diseased. The 40 plants were classified into healthy (YTH) and diseased (YTD) groups based on disease investigation results. Soil samples from seven healthy tobacco plants were pairwise mixed to form three replicates for the YTH group on days 7, 14, 21, and 28, respectively, and one plant soil sample was obsoleted. Similarly, soil samples from 33 diseased tobacco plants were equally divided into three parts, each of which was mixed to form three replicates for the YTD group. Finally, each group had three replicated soil samples on days 7, 14, 21, and 28, respectively, for the subsequent detection of the soil bacterial community succession and soil physicochemical properties.

### Measurement of soil physicochemical properties

The soil physicochemical properties were measured as described in our previous study (Wang et al., 2024). The soil pH was determined by a pH meter. Soil organic matter content was determined by the potassium dichromate oxidation method. The soil AP content was determined by the molybdenum antimony resistance colorimetric method, the soil AK content was determined by flame photometer, the content of NH_4_^+^-N and NO_3_^–^-N were determined by ultraviolet spectrophotometry, and the soil AP content was determined by sulfuric acid-accelerator digestion. The TN content was determined by the Kjeller method, the TP content was determined by NaOH alkali melting and molybdenum antimony resistance spectrophotometry, and soil TK content was determined by NaOH alkali melting and flame photometer.

### Soil DNA extraction, amplification, 16S rRNA gene sequencing, and data processing

DNA extraction, amplification, 16S rRNA gene amplicon sequencing, and data processing were described in detail in our previous study (Xiao et al., 2018). The PowerSoil DNA Isolation Kit (TIANGEN BIOTECH, Beijing, China) was used to isolate DNA from the soils. DNA extracts were purified by electrophoresis on a 1% agarose gel using a DNA gel extraction kit (OMEGA, USA) and the concentration was measured by a Nanodrop 2000 microspectrophotometer (NanoDrop Technologies, Wilmington, NC, USA). The 16S rRNA gene was amplified with primer pair 338F (5′-CTCCTACGGGAGGCAGCA-3′) and 806R (5′-GGACTACHVGGGTWTCTAAT-3′). PCR products were purified, quantified, and homogenized to construct the library. The products were sequenced on an Illumina Novaseq machine (Illumina, San Diego, CA, USA). The raw data of 16S rRNA gene sequences was deposited in the Sequence Read Archive (SRA) of NCBI under the accession number PRJNA1021354.

Pair-end reads were processed using Trimmomatic v0.33 to detect and remove the sequences with a quality score below 20. Sequences were denoised and chimera filtered with the UCHIME v4.2 to obtain the effective reads, which were further clustered into operational taxonomic units (OTUs) using a 97% identity threshold. The taxonomic assignment used an RDP classifier with a minimal 50% confidence estimate.

### Molecular ecological network construction and characterization

Random matrix theory (RMT)-based approaches were used for network construction (Xiao et al., 2018), hub and connector OTU identification and the topological property were determined with a similar threshold (0.96). OTUs, presented in 12 out of 12 replicates, were used for network analysis to ensure data reliability. Various network properties were characterized, such as average degree, average path distance, average clustering coefficient, and modularity index. The network modules were generated using rapid greedy modularity optimization. The experimental data used for constructing phylogenetic molecular ecological networks (pMENs) were based on 16S rRNA gene sequences, and Gephi 0.9.2 software was used to visualize network graphs. The pMENs were constructed separately based on sequencing data of two treatments to reveal the differences between the soil bacterial network interactions of healthy and diseased plants.

### Functional profiling

Prior to functional gene prediction using PICRUSt (phylogenetic investigation of communities by reconstruction of unobserved states) described by Langille et al. (2013), the detected OTUs were reclassified using the GREENGENES reference database. Subsequently, PICRUSt uses 16S rRNA genes to infer metagenome gene functional content from phylogenetic information. The predictions are precalculated for genes in databases, including the Kyoto Encyclopedia of Genes and Genomes (KEGG). The input data were first normalized by copy number by dividing each OTU by the known 16S rRNA copy number abundance before metagenome predictions and subsequent collapse into functional pathways. The output of PICRUSt consists of a table of functional gene counts as KEGG orthologs (KOs). The Nearest Sequenced Taxon Index (NSTI) value was used to validate the reliability of predicted metagenomes and functional pathways.

### Statistical analysis

The community diversity was assessed using the number of OTUs, Shannon diversity index (*H*’), Chao 1, and Simpson evenness index. Differences in diversity and relative abundances of bacterial composition based on Tukey’s test were conducted by a one-way analysis of variance (ANOVA) (Deng et al., 2012). Principal Coordinates Analysis (PCoA) was conducted to analyze the bacterial community structure. LDA Effect Size (LEfse) analysis was conducted to find the species with significant differences between groups (Barberán et al., 2014; Wagg et al., 2014; Cui et al., 2016). All analyses were performed using R v.3.6.3 and on Tutools.^1^

## Results

### TMV incidence and soil physicochemical properties

**FIGURE 1:**
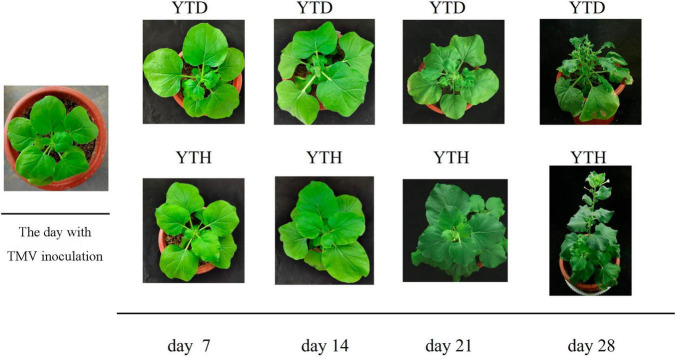
The incidence of *N. benthamiana* in YTD and YTH groups at different stages (day 7, 14, 21, and 28).

**FIGURE 2:**
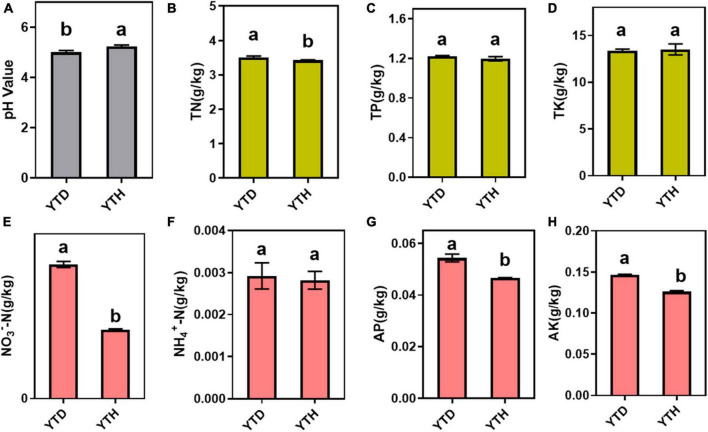
Soil physicochemical properties, including pH value **(A)** and the total contents of total nitrogen **(B)**, total phosphorus **(C)**, total potassium **(D)**, NO_3_^–^-N **(E)**, NH_4_^+^-N **(F)**, available phosphorus **(G)**, and available potassium **(H)**. Differences in soil physicochemical properties based on Tukey’s test were conducted by ANOVA (*n* = 3). Different lowercase letters represent significant differences between groups at the 5% level.

The incidence of *N. benthamiana* in the YTD and YTH groups at different stages is shown in Figure 1. Compared with healthy tobacco in the YTH group all the time, tobacco leaves curled on day 7 and then gradually became severe in the YTD group after TMV inoculation. According to the results of soil physicochemical properties on day 28 (Figure 2), we found the pH value (YTD: 5.01; YTH: 5.25) was significantly lower, and the contents of TN (YTD: 3.51 g/kg; YTH: 3.43 g/kg), NO_3_^–^-N (YTD: 0.045 g/kg; YTH: 0.023 g/kg), AP (YTD: 0.054 g/kg; YTH: 0.047 g/kg), and AK (YTD: 0.147 g/kg; YTH: 0.126 g/kg) were significantly higher in the YTD group than in the YTH group. These results suggested that tobacco plants grew better in the YTH group than in the YTD group.

### Overview of soil bacterial communities

**FIGURE 3:**
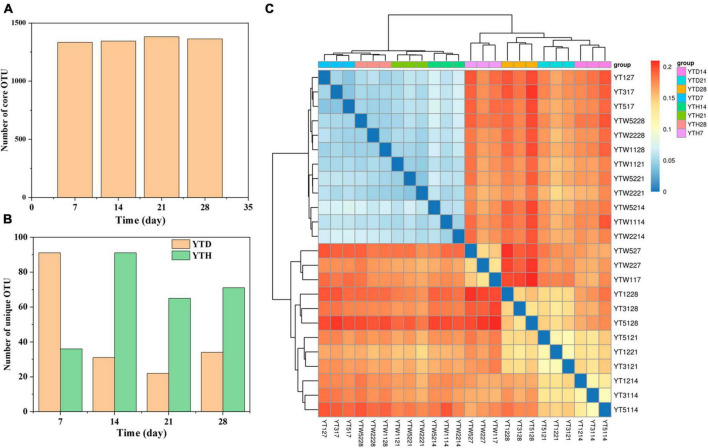
The number of core OTU **(A)** and unique OTU **(B)** in the YTD and YTH groups, and **(C)** heatmap analysis used Bray-Curtis dissimilarity metrics of bacterial community at OTU level. The number of core and unique OTU were statistic based on the 12 samples of each group.

A total of 1,518,787 quality sequences were obtained, with between 60,756 and 65,938 sequences per sample. After clustering, 1473 OTUs were detected in all samples. Among them (Figure 3A), the number of core OTUs was the highest on day 21 (1382) and the lowest on day 7 (1335). The number of unique OTU in the YTD group was higher on day 7 and more down on days 14, 21, and 28 than that in the YTH group (Figure 3B). Heatmap analysis of the bacterial community showed that samples in YTH on day 7 were clustered with the samples in YTD on day 14, 21, and 28. In contrast, samples in YTD on day 7 were clustered with the samples in YTH on days 14, 21, and 28 (Figure 3C).

**FIGURE 4:**
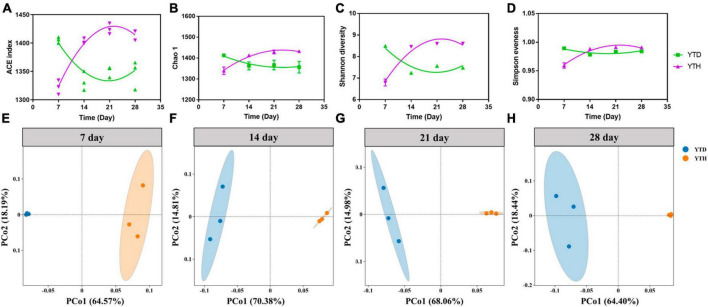
The change of diversity indices [**(A)** number of OTU; **(B)** Chao 1; **(C)** Shannon diversity; **(D)** Simpson evenness] and the ordination plots of all samples for the community structure analyzed by PCoA on day 5 **(E)**, 14 **(F)**, 21 **(G)**, and 28 **(H)**, respectively. Non-linear fitting was conducted in Graphpad software (version 8.0) to analysis the change trend of diversity indices.

The α-diversity indices, including the number of OTUs, Chao 1, Shannon diversity, and Simpson evenness, were shown in Figures 4A–D. After inoculating TMV, the α-diversity indices were significantly higher in the YTD group than in the YTH group on day 7. However, the diversity indices were increased constantly in the YTH group and decreased in the YTD group, resulting in the diversity indices being significantly higher in the YTH group than those in the YTD group on days 14, 21, and 28.

Principal Coordinates Analysis results showed that the YTH samples were segregated from the YTD samples during the whole planting stage (Figures 4E–H). Additionally, samples within the YTD group were clustered more closely on day 7 than on other days, and it was interesting that samples within the YTH group were clustered more closely on days 14, 21, and 28 than on day 7.

### The compositions of soil bacterial communities

**FIGURE 5:**
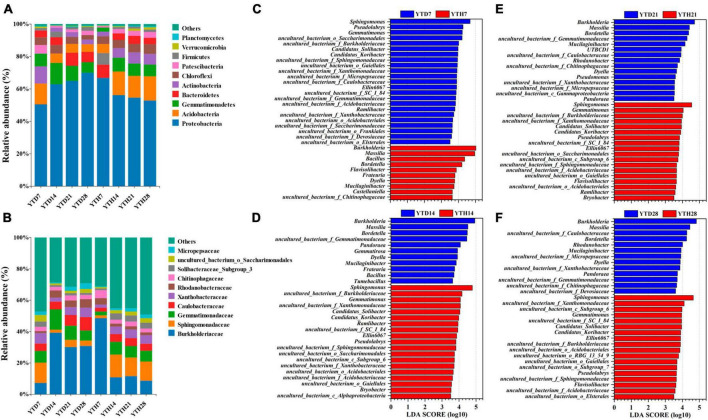
The compositions of bacterial communities in phylum **(A)**, family level **(B)**, and the significant genera between YTD and YTH groups according to LEfSe analysis on day 7 **(C)**, 14 **(D)**, 21 **(E)**, and 28 **(F)**, respectively.

After inoculating TMV, soil bacterial community compositions were explored and analyzed at four-time points. At the phylum level (Figure 5A), the bacterial composition consisted of 21 phyla. The dominant phyla (top 6) included Proteobacteria (50.34–70.99%), Acidobacteria (4.59–15.06%), Gemmatimonadetes (2.43–13.31%), Bacteroidetes (3.74–8.78%), Actinobacteria (2.41–10.67%) and Chloroflexi (2.59–5.98%), etc. At the family level (Figure 5B), 217 families were found among all soil samples, and the dominant families (top 6) were Burkholderiaceae (7.08–51.31%), Sphingomonadaceae (1.71–14.84%), Gemmatimonadaceae (2.26–13.10%), Caulobacteraceae (2.37–8.63%), Xanthobacteraceae (1.78–6.67%), Rhodanobacteraceae (1.21–5.67%), etc. In addition, all 1473 OTUs were affiliated to 345 genera, and the dominant genera (relative abundance >1%) included *Burkholderia*, *Massilia*, *Sphingomonas*, *Bordetella*, *Mucilaginibacter*, *Gemmatimonas*, *Bacillus*, *Pseudolabrys*, *Flavisolibacter*, *Ramlibacter*, *Phenylobacterium*, *Dyella*, *Rhodanobacter*, *Gemmatirosa*, *Pandoraea*, and *Ellin6067*.

To further explore the differences between the soil bacterial community compositions of healthy and diseased plants, LEfse analysis at the genus level was conducted, and LDA analysis showed the main genera with significant differences (Figures 5C–F). The findings were as follows: on day 7, the principal genera in YTD were *Sphingomonas*, *Pseudolabrys*, and *Gemmatimonas*, and in YTH were *Burkholderia*, *Massilia*, and *Bacillus*; on day 14, 21, and 28, it was contrary that the main genera in YTD were *Burkholderia*, *Massilia*, *Bordetella* and *Dyella*, and in YTH were *Sphingomonas*, *Gemmatimonas*, *Ellin6067* and *Pseudolabrys*.

### Network interactions of bacterial communities in healthy and diseased soil

**FIGURE 6:**
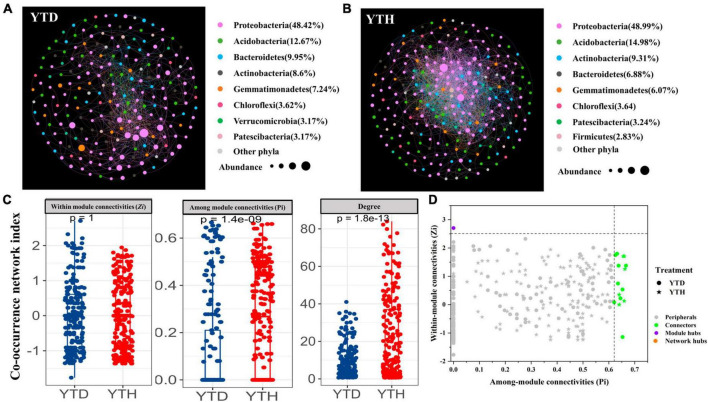
Molecular ecological networks in YTD **(A)**, and YTH **(B)** groups, the co-occurrence network index **(C)**, and the Z-P plot showing the distribution of OTUs based on their topological roles **(D)**. The color of the circle represented the module in the network interaction. The topological role of each OTU was determined according to the scatter plot of within-module connectivity (Zi) and among-module connectivity (Pi).

We constructed bacterial networks to describe the symbiotic interactions at OTU level to better understand the interrelationships of soil bacterial communities in the healthy and diseased plants after inoculating TMV. The network nodes were mainly divided into Proteobacteria, Acidobacteria, Bacteroidetes, Actinobacteria, and Gemmatimonadetes (Figures 6A, B). The relative abundances of total nodes were about 63.44 and 69.38%, respectively, in the YTH and YTD groups. Further analysis found that the proportion of *Sphingomonas* (13.97%), *Burkholderia* (10.29%), and *Massilia* (9.11%) were the three genera with the enormous proportions in YTH group, which were different from that of *Burkholderia* (18.22%), *Massilia* (8.19%) and uncultured Gemmatimonadaceae (6.80%) in the YTD group.

Major topological parameters of empirical MENs of bacterial communities in the two groups revealed that the soil bacterial community network of the YTD group was simpler than that of the YTH group (Table 1). With the same threshold (0.960), their correlations were more than 0.500, indicating that the degree distributions in both constructed molecular ecological networks fitted the power-law model well. There were more nodes and links in the YTH group (247 nodes and 2571 links) than in the YTD (221 and 947) group. The healthy network significantly increased the *Zi*, *Pi*, and degree, which indicated the creation of more intricate network patterns (Figure 6C). It showed that TMV might disrupt interactions of soil bacterial communities, and simpler eco-network showed weaker resistance to TMV, causing disease in the tobacco plant.

**TABLE 1:** Topological properties of the empirical pMENs of soil bacterial communities in two groups.

Network indexes	YTD	YTH
Total nodes	221	247
Total links	947	2571
Positive links	578 (61.03%)	1512 (58.81%)
Negative links	369 (38.97%)	1059 (41.19%)
Threshold	0.960	0.960
Module	14	21
Modularity	0.462	0.272
R square of power-law	0.707	0.499
Average degree (avgK)	8.57	20.818
Average clustering coefficient (avgCC)	0.421	0.444
Average path distance (GD)	4.032	2.839
Geodesic efficiency	0.329	0.43
Harmonic geodesic distance (HD)	3.042	2.328
Maximal degree	41	84
Nodes with max degree	OTU39	OTU5
Centralization of degree (CD)	0.149	0.259
Maximal betweenness	2870.714	1142.729
Nodes with max betweenness	OTU20	OTU5
Centralization of betweenness (CB)	0.111	0.032
Maximal stress centrality	381249	38698
Nodes with max stress centrality	OTU20	OTU74
Centralization of stress centrality (CS)	15.027	1.062
Maximal eigenvector centrality	0.239	0.17
Nodes with max eigenvector centrality	OTU39	OTU107
Centralization of eigenvector centrality (CE)	0.206	0.13
Density (D)	0.039	0.085
Transitivity (Trans)	0.503	0.569
Connectedness (Con)	0.635	0.801
Efficiency	0.945	0.899

We classified nodes into four categories, including peripherals, connectors, module hubs, and network hubs. Connectors, module hubs, and network hubs are commonly considered to be the keystone in the co-occurrence network. The results found that there were also more module hubs (1 hub) and connectors (9 connectors) in the YTD group than those in the YTH group (Figure 6D). The keystone genera in the YTH group were classified as *Oryzihumus*, *Niastella*, and other unknown genera. The keystone families were classified as Sphingomonadaceae, Xanthobacteraceae, Intrasporangiaceae, Chitinophagaceae, and SC-I-84 (Table 2). However, with the TMV outburst, the keystone genera in the YTD group were replaced by *Burkholderia*, *Massilia*, *Rhodanobacter*, and *Koribacter*, as well as other unknown genera, and the keystone families were Burkholderiaceae, Xanthobacteraceae, Rhodanobacteraceae and Koribacteraceae (Table 2).

**TABLE 2:** Hubs in the molecular ecological networks.

Group	Hubs	OTU	Phylum	Family	Genus	Average relative abundance (%)
YTD	Module hubs	OTU20	Proteobacteria	Burkholderiaceae	*Burkholderia*	1.657
	Connectors	OTU7	Proteobacteria	Burkholderiaceae	*Burkholderia*	2.124
		OTU520	Proteobacteria	Burkholderiaceae	*Massilia*	0.288
		OTU39	Proteobacteria	Burkholderiaceae	*Burkholderia*	1.398
		OTU342	Proteobacteria	Burkholderiaceae	Unknown genus	0.217
		OTU142	Actinobacteria	Unknown family	Unknown genus	0.082
		OTU2553	Proteobacteria	Rhodanobacteraceae	*Rhodanobacter*	0.335
		OTU54	Acidobacteria	Koribacteraceae	*Koribacter*	0.249
		OTU7691	Proteobacteria	Burkholderiaceae	Unknown genus	0.058
		OTU4417	Proteobacteria	Xanthobacteraceae	Unknown genus	0.097
YTH	Connectors	OTU35	Proteobacteria	Sphingomonadaceae	Unknown genus	0.215
		OTU41	Patescibacteria	Unknown family	Unknown genus	0.155
		OTU31	Proteobacteria	Xanthobacteraceae	Unknown genus	0.391
		OTU153	Actinobacteria	Intrasporangiaceae	*Oryzihumus*	0.059
		OTU381	Bacteroidetes	Chitinophagaceae	*Niastella*	0.034
		OTU346	Proteobacteria	SC-I-84	Unknown genus	0.020

### The succession of key genera or families with inoculating TMV

**FIGURE 7:**
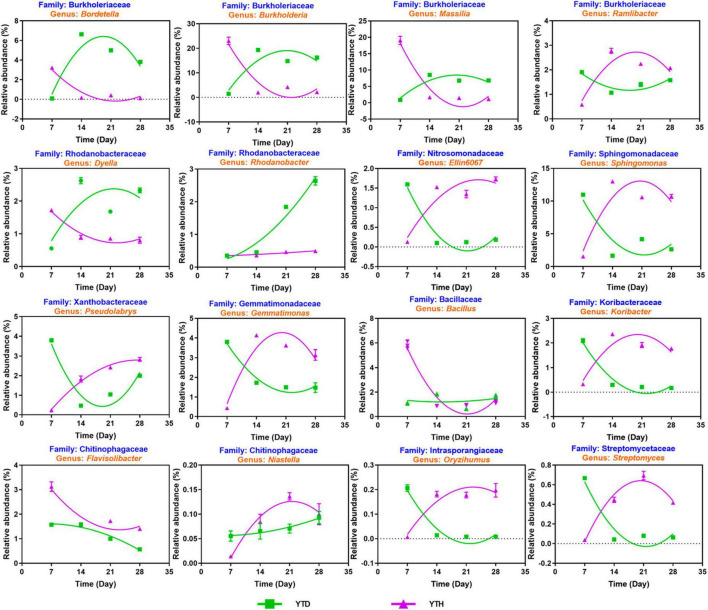
The succession of 16 key genera after inoculating TMV in the YTD and YTH groups by non-linear fitting in Graphpad software (version 8.0).

Based on the above analysis, we explored the succession of sixteen possible key genera (Figure 7). Among these genera, four including *Bordetella*, *Burkholderia*, *Massilia*, and *Dyella*, showed a similar succession tendency, that is, the relative abundances were higher in YTD than those in YTH group on day 7, while were decreased gradually in YTD and were progressively increased in YTH, resulting in that higher in YTH on the later time points. The succession of Nine genera, including *Ramlibacter*, *Elin6067*, *Sphingomonas*, *Pseudolabrys*, *Gemmatimonas*, *Koribacter*, *Niastella*, *Oryzihumus*, and *Streptomyces*, was an opposite tendency with the above four genera. The relative abundance of *Rhodanobacter* gradually increased in the YTH group and changed a little in YTD. The relative abundance of *Bacillus* decreased quickly on day 14 in the YTD group and changed slightly in YTH. The relative abundance of *Flavisolibacter* gradually reduced in both groups and was higher in the YTD group.

### Metabolic diversity

**FIGURE 8:**
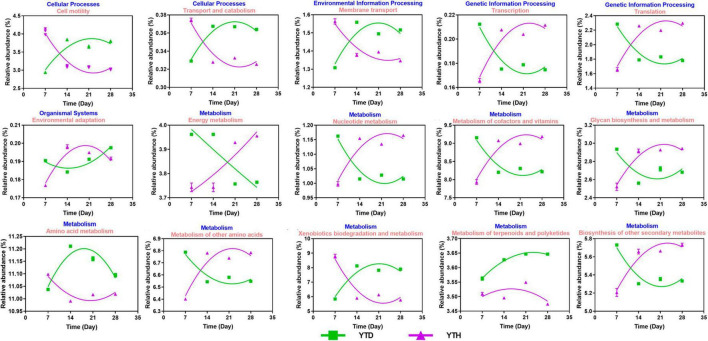
The change tendency of predicted metabolic pathways after inoculating TMV in the YTD and YTH groups by non-linear fitting in Graphpad software (version 8.0).

Based on the 16S rRNA sequences, potential functions were predicted to explore the differences in soil bacterial functional genes between the YTH and YTD groups (Figure 8). It indicated that the relative abundance of genes changed considerably in the early stage (day 7 and 14) after TMV inoculated. The relative abundances of genes related to Transcription, Translation, environmental adaption, and metabolism (nucleotide, cofactors and vitamins, Glycan biosynthesis, other amino and other secondary metabolites) showed higher in the YTH group than that in the YTD group on day 7 while it was on the contrary on day 14. The relative abundances of cell mobility, transport and catabolism, membrane transport, and metabolism (amino acid and xenobiotics biodegradation) showed higher in the YTD group than in the YTH group on day 7, while it was on the contrary on day 14.

## Discussion

Previous studies focused on isolating and applying beneficial microbes in controlling plant pathogens (Dunne et al., 1997; Liu et al., 2007; Shanks et al., 2012; Han et al., 2016). However, it is also essential to understand the response of plants and their soil microbial community when faced with pathogen infection. Research on plant-microbe interactions revealed that plants can shape their rhizosphere microbiome. Recent advances in pathogen or insect attacks show that plants can recruit protective microorganisms and enhance microbial activity to suppress pathogens (Berendsen et al., 2012; Stringlis et al., 2018). In this study, we studied which bacteria could be recruited by *N. benthamiana* and explored the possible mechanism of plant-bacteria interaction to respond to TMV attack.

This study found that there was significant variation in the bacteria community between the two groups of treatments. The network has become more complex in YTH, which is conducive to strengthening the stability and function of bacterial communities (Hernandez et al., 2021). At the same time, the proportion of beneficial bacteria (e.g., *Ramlibacter*, *Sphingomonas*, *Streptomyces*, and *Niastella*) in YTH shows an increasing trend compared to YTD. *Ramlibacter* and *Sphingomonas* can produce extracellular polysaccharides, which are beneficial for activating defense-related genes and enhancing the activity of defense-related enzymes to enhance plant disease resistance (Meneghine et al., 2017; Canwei et al., 2020; Jivkova et al., 2022). *Streptomyces* can improve the activity of plant antioxidant enzymes (SOD, CAT, POD, and MDA), assisting plants in resisting viral infections (Chen et al., 2022). Ara et al. (2012) found streptomyces derived from various bioactive compounds to effectively minimize the TMV local lesions on the leaves of weed plants. *Niastella* can promote plant growth and defense against pathogens (Cuartero et al., 2022). Overall, the beneficial microorganisms were consistent with the previous studies (Manjunatha et al., 2022).

In addition, most metabolic activities of bacteria in the two groups of treatments also showed an opposite variation trend. The enhancement of the genetic information process (Transcription and translation) and metabolism (nucleotide, glycan biosynthesis, and other amino acids) were crucial for bacterial growth, reproduction, and environment adaptability in YTH. Glycan and its compounds, e.g., mannosyl erythritol lipids (Cortés-Sánchez et al., 2013) and glycoprotein (Zhang et al., 2015), displayed promising adequate substitutes for biocontrol agents for many applications in agricultural production. Many studies found that amino acid metabolic pathways could regulate plant immunity (Zeier, 2013). Proline metabolism is involved in the oxidative burst and the hypersensitive response associated with pathogen recognition (Chen et al., 2011; Zeier, 2013). Asp-derived pyridine nucleotides influence invasion immunity by modulating the crosstalk of salicylic acid- and jasmonic acid-regulated defense pathways (Zhang and Mou, 2009).

Remarkably, we discovered that the bacteria community diversity, the proportion of beneficial bacteria, and bacteria metabolic levels of YTH were lower than YTD on day 7 in this study. However, on the 14th day, the opposite phenomenon was observed, YTH higher than YTD and remaining stable afterward. Based on the results of PCoA and bacterial succession, we propose a mechanism of soil bacterial community and *N. benthamiana* response to TMV infection. The reagency or sensibility of the soil bacterial community might influence the response of *N. benthamiana* to TMV attack. If the soil bacterial community is insensitive or sluggish, viral activity leads to bacterial lysis, facilitating the release of signal molecules that are subsequently transmitted to tobacco plants. The threatened tobacco plants activate their intrinsic defense mechanisms and excrete metabolites, leading to recruiting the beneficial bacteria. This restructuring includes increased helpful bacteria to combat the viral invasion, subsequently protecting the plant. However, suppose the soil bacterial community is sensitive or responds rapidly. In that case, some bacterial strains exhibit specific resistance to virus invasion, thereby impeding the prompt transmission of signal molecules to tobacco plants. Consequently, the activation of tobacco defense mechanisms is hindered, resulting in a rapid increase of TMV virosome. This abrupt rise in TMV levels disrupts the structure of the bacterial community and reduces the proportion of beneficial bacteria. Ultimately, these alterations in the microbial community significantly impact tobacco growth. It is also explained that it was similar between the soil bacterial communities in the YTH group disrupted by TMV on day 7 and in the YTD group disrupted by TMV on days 14, 21 and 28 (Figure 3C).

Further investigation is warranted to elucidate the precise response mechanisms of tobacco to soil TMV. Additionally, this study conducted a comparative analysis of the bacteria community present in various tobacco soils, identifying several potentially beneficial bacteria that hold promise for future research endeavors.

## Conclusion

With TMV inoculated, although the alpha diversity of the bacterial community in the YTD was markedly higher at the early stage, it subsequently decreased to lower than in the YTH group. Ecological network was also more complexed in the YTH than in the YTD group. In addition, there was an increase of beneficial bacterial (e.g., *Ramlibacter*, *Sphingomonas*, *Streptomyces*, and *Niastella*) in the YTH group, which might contribute to strengthening resistance of Tobacco to TMV.

## Data availability statement

The datasets presented in this study can be found in online repositories. The names of the repository/repositories and accession number(s) can be found below: https://www.ncbi.nlm.nih.gov/genbank/, PRJNA1021354.

## Author contributions

YZ: Data curation, Formal analysis, Investigation, Methodology, Writing – review and editing. TL: Data curation, Investigation, Project administration, Resources, Supervision, Writing – review and editing. SW: Data curation, Investigation, Writing – review and editing. DZ: Funding acquisition, Supervision, Writing – review and editing. ZX: Data curation, Writing – review and editing. ZR: Resources, Writing – review and editing. LL: Data curation, Writing – review and editing. SL: Data curation, Writing – review and editing. YX: Data curation, Formal analysis, Visualization, Writing – original draft. QT: Conceptualization, Formal analysis, Funding acquisition, Supervision, Visualization, Writing – review and editing.

## Footnotes

1
http://www.cloudtutu.com
